# *Klebsiella michiganensis*: a nitrogen-fixing endohyphal bacterium from *Ustilago maydis*

**DOI:** 10.1186/s13568-023-01618-8

**Published:** 2023-12-19

**Authors:** Pengyu Liang, Jianwei Jiang, Zhengxiang Sun, Yanyan Li, Chunlei Yang, Yi Zhou

**Affiliations:** 1 Department of Plant Protection, College of Agriculture, Yangtze University, Jingzhou, 434025 China; 2 Tobacco Research Institute of Hubei Province, Wuhan, 430000 China

**Keywords:** Endohyphal bacterium, *Klebsiella michiganensis*, Nitrogen fixation, *Ustilago maydis*

## Abstract

*Ustilago maydis* is a pathogenic fungus in *Basidiomycota* causing corn smut disease. A strain of *U. maydis* YZZF202006 was isolated from the tumor of corn smut collected from Jingzhou city in China. The intracellular bacteria were confirmed inner hyphal of the strain YZZF202006 by PCR amplification and fluorescence in situ hybridization (FISH) and SYTO-9. An endohyphal bacterium YZUMF202001 was isolated from the protoplasts of the strain YZZF202006. It was gram-negative, short rod-shaped with smooth light yellow colony. The endohyphal bacterium was genomic evidenced as *Klebsiella michiganensis* on the basis of average nucleotide identity (ANI) analysis and the phylogram. Then *K. michiganensis* was GFP-Labeled and reintroduced into *U. maydis*, which confirmed the bacterium can live in hyphae of *U.maydis*. The bacterium can grow on N-free culture media. Its nitrogenase activity was reached av. 646.25 ± 38.61 nmol·mL^− 1^·h^− 1^ C_2_H_4_ by acetylene reduction assay. A cluster of nitrogen fixation genes (*nifJHDKTXENXUSVWZMFLABQ*) was found from its genome. The endohyphal *K. michiganensis* may play an important role to help nitrogen fixation for fungi in the future.

**Supplementary Information:**

The online version contains supplementary material available at 10.1186/s13568-023-01618-8.

## Key points

A endohyphal bacterium *Klebsiella* sp. was found and isolated from *Ustilago maydis.*

Endohyphal bacterium was identified and evaluated for nitrogen fixation.

A GFP-Labeled *K. michiganensis* was reintroduced into *U. maydis* by a new method.

**Supplementary Information:**

The online version contains supplementary material available at 10.1186/s13568-023-01618-8.

## Introduction

Nitrogen is the most abundant element in atmosphere, considered as a constituent of protein, nucleic acid and other essential molecules in organism. Although nitrogen is highly stored in the air, it can’t be directly utilized by most organisms (Beringer and Hirsch 1984). The nitrogen in the air requires a complex fixing process to be used by the biosome. There are three main ways for the transforming, including the fixation under high temperature or lightning conditions, the industrial Haber Bosch process producing needed nitrogen fertilizers, and biological nitrogen fixation (NF). The biological NF involves the reduction of dinitrogen (N_2_) to ammonia (NH_3_), available for the utilization of most organisms, which is a typical example of the inefficiency of industrial NF processes (Tsujimoto et al. 2016; Cherkasov et al. 2015).

The discovery of endohyphal bacteria (EHB) has been going on for half a century. EHB exist within fungi and are a part of their entire, playing their own functions and even having an impact on its host fungi. Many impacts have been reported, including the morphology and growth of hyphae, enzyme activity, toxins from pathogenic fungi, nitrogen fixation, indole-3-acetic acid (IAA) related to growth promotion, and mycorrhizal formation and so on. EHB can increase the spore production of host fungi, control their spore production ability, and promote fungal growth (Lumini et al. 2007; Araldi-Brondolo et al. 2017; Pakvaz & Soltani 2016; Guo et al. 2017). But there are also opposite examples, after removing the EHB of the pathogenic fungus *Rhizopus microsporus* of rice, it cannot grow and produce spores normally (Partida-Martinez et al. 2007). The presence of EHB *Luteibacter* sp. promotes the growth of its host fungi, *Pestalotiopsis* sp. and *Microdiplodia* sp (Arendt et al., 2015b). EHB can directly affect cellulase activity or indirectly affect lignin enzyme activity (Arendt et al., 2015a). The true reason for the production of rhizoxin, which is caused by the pathogen of rice seedling wilt disease, is the EHB *Paraburkholderia rhizoxinica* (Partida-Martinez & Hertweck 2005). *Pestalotiopsis* sp. and its EHB *Luteibacter* sp., *Fusarium oxysporum*, and its EHB *Klebsiella aerogenes* are all related to the synthesis of host fungi IAA (Hoffman et al. 2013; Cheng et al. 2022). There are many interactions between fungi and their EHB as mentioned above, among which NF is an important type. Because the nitrogen reduction of biological NF is catalyzed by nitrogenase system, however nitrogenase only exists in some prokaryotes, not in fungi (Rubio and Ludden 2005; Hu et al. 2008), it can serve as a supplement to fungal function. A *Basidiomycete Rhodotorula mucilaginosa* can grow on medium without nitrogen by harboring *Pseudomonas stutzeri* with NF in hyphal cells (Paul et al. 2020). Three ‘probable endobacteria’ isolated from the spores of arbuscular mycorrhizal fungi (*Glomeromycota*) exhibit the activity of nitrogenase (Cruz and Ishii 2012). Tuber-associated NF bacteria of the well-known Italian white truffle (*Tuber magnatum*, *Ascomycetes*) have been determined as *Bradyrhizobium* spp. (Barbieri et al. 2010). These examples strongly support the view that the NF function of EHB can serve as a supplement to fungal function.

Although bacteria can live in fungal cells that have been found for more than half a century (Mosse 1970), there are just few reports about endohyphal bacteria with NF. Recently, *Ustilago maydis*, a causal agent of corn smut in *Basidiomycota* can be able to grow normally in N-free medium and evidenced containing NF endohyphal bacteria of *Bacillus* spp. by microscopic observations and PCR amplification (Ruiz-Herrera et al. 2015). However, the endohyphal bacteria could not be successfully isolated. Therefore, the aim of this study is to isolate EHB with NF ability from *Ustilago maydis* that can grow in a N-free environment. On the one hand, it is to verify that there are indeed EHB with NF in its hyphal. On the other hand, it is to quantitatively detect the nitrogenase activity of EHB, determine its NF ability, and use these as materials to conduct research on the interaction between host fungi and EHB. In 2020, a strain YZZF202006 of *U. maydis*, was obtained from corn smut tumor from Jingzhou city of China, which could grow on N-free medium. This study was conducted to examine the existence of endohyphal bacteria with fluorescence in situ hybridization (FISH) and PCR amplification of partial fragments of 16s rDNA. Then, the isolated EHB strain YZUMF202001 was identified by genomic information, and its NF ability was evaluated.

## Materials and methods

### EHB examination in *Ustilago maydis*

In June of 2020, tumors of corn smut were collected in corn field in Taihu Town of Jingzhou, Hubei Province, China. The tumor was surface sterilized with 75% ethanol for 30s and sodium hypochlorite (NaOCl) for 2 min, and rinsed for three times by sterile distilled water. The tissues was grounded and diluted spreading on yeast extract peptone sucrose light (YEPSL) media (0.4% yeast extract, 0.4% peptone, 2% sucrose and 2% agar) to collect the strains of *U. maydis*. The obtained strains were screened by FISH to detect the existence of EHB (Takashima et al. 2018; Ruiz-Herrera et al. 2015). Among them, one strain YZZF202006 was substantially observed during the sub-culturing, which was verified as *U. maydis* based on morphology and phylogenetic analysis of ITS region (White et al. 1990) (Figure S1).

For FISH, the sample was fixed in 500 µL 4% formalin, dissolved in phosphate buffered saline (PBS), placed at 4 °C for 3 h, washed twice with 500 µL PBS solution in 1.5 mL centrifuge tube, and then dehydrated in solutions of anhydrous ethanol (50%, 70% and 95%) for 3 min. The resulted sample was hybridized with an oligonucleotide probe solution containing the general bacterial 16 S rRNA gene probe EUB338 (Takashima et al. 2018), which was labeled at the 5ʹ-end with Cy3 (red) or FAM (green). For hybridization, 100 µL of hybridization solution (40% formamide, 35% DEPC-Treated Water, 25% EDTA and 0.01 µM oligonucleotide probe) was added and kept in dark at 46 °C for 1.5 h. The sample was immediately washed twice with washing buffer (NaCl 50 mM, SDS 0.01%, Tris-HCl 20 mM, pH = 7.2). In the end, it was placed on slide and photographed under a fluorescence microscope equipped with Nikon DS-Ri2 photography system (Nikon, Japan).

Refer to the instructions of STYO-9 Green Fluorescent Nuclear Acid Stains for experiments. Inoculate the fungus YZZF202006 into YEPSL medium, culture it under 28 ℃ for 48 h, rinse it with 8.5 g/L NaCl for three times, add 100 µL 8.5 g/L NaCl to gently suspend the cell, then add 100 µL 10 µmol/L SYTO-9 green fluorescent nucleic acid stain, mix it well, incubate it in the dark for 15 to 30 min and observe it.

In order to know the status of endohyphal bacterium of *U. maydis*, a primer pair of 16 S rDNA region with a 650 bp target fragment, 16sU1f (5’-GGGATAACTACTGGAAACGG-3’) and 16sU1r (5’-CCACTCCTCAAGGGAACAA-3’), was designed by software Primer Premier 5 (Lalitha 2000). The genomic DNA of strain YZZF202006 was exacted from colonies grown nitrogen-free medium for 3 days by CTAB method (Stenglein and Balatti 2006). Polymerase chain reaction (PCR) amplification was conducted with primers of 16sU1f and 16sU1r. A 50 µL of the PCR reaction mixture comprising 25 µL of 2 × Taq Master Mix (Vazyme, Nanjing, China), 4 µL template DNA, 2.5 µL of each primer and 16 µL ddH_2_O was applied and performed in a BIORAD T100 thermocycler (Bio-Rad, USA). PCR was conducted by using the following steps: pre-denaturation at 95 ℃ for 3 min, 35 cycles of denaturation at 95 ℃ for 15s, annealing at 52 ℃ for 15s, extension at 72 ℃ for 60s, with a final extension at 72 ℃ for 10 min. Amplified PCR products were purified and sequenced by TSINGKE company (Beijing, China). The resulted sequence was primarily compared by BLASTn algorithm in GenBank database. The reference sequences used in the phylogenetic analysis were retrieved from the LSPN (https://www.bacterio.net/) and downloaded from the GenBank database. The phylogenetic tree was constructed using Maximum-Likelihood (ML) method in MEGA v.7.0.26 (Kumar et al. 2016). Bootstrap consensus values were calculated using 1,000 replicates. Branch support values above 60% were shown at the nodes in the phylogram.

### EHB isolation from *Ustilago maydis*

The hyphae of *U. maydis* were inoculated into YEPSL liquid media at 28 ℃ with constant shaking (150 rpm) for 2 days. A volume of 1 mL liquid (around 10^6^ spores / mL) was transferred in new fresh YEPSL media cultured for 12 h under the same conditions. Then fresh spores were harvested by centrifugation (7,000 rpm, 10 min), washed twice in 10 mL 0.8 M NaCl solution, re-suspended in 20 mL of lysis solution (A mixture of 20 mg / mL Driselase and 20 mg / mL lyticase filtrated by 0.22 μm microporous filter), and shaken (75 rpm) at 28 ℃ for 3 h. Protoplasts were collected by centrifugation (4,000 rpm, 10 min) and washed twice with 10 mL STC solution (1 M sorbitol, 10 mM Tris-HCl, 50 mM CaCl_2_, pH = 7.5). The spore pellet was re-suspended in 2 mL STC solution and put into a sterile mortar containing appropriate amount of sterile quartz sand for grinding. The grinded solution (100 µL) was spread on a nitrogen-free culture medium (Hopebio Company, Qingdao, China) and incubated at 28 ℃. The whole process of EHB isolation is completed under sterile conditions. The isolation was repeated for two times. The uniform colonies from nitrogen-free plates were arising and pure strains were preserved by obtaining single colony.

### Identification of strain YZUMF202001 from *Ustilago maydis*

One representative strain YZUMF202001 was randomly selected for the identification. It was streak cultured on Luria-Bertani (Sezonov et al. 2007) plate (10 g tryptone, 5 g yeast extract, 10 g NaCl, 15 g agar, 1 L distilled water) at 28 ℃ for 2 days to observe the colony morphology. To determine the bacterial cell morphology, the EHB cells were collected and fixed in 2.5% glutaraldehyde solution for 4 h and rinsed three times for 10 min with phosphate buffer (0.2 M, pH = 7.4). The samples were then dehydrated in a series of ethanol solutions for 15 min: 30% (once), 50% (once), 70% (once), 85% (once), 95% (once) and 100% (twice) and washed in isoamyl acetate for twice 15 min. To form blocks, samples were processed in a vacuum freeze dryer for 3 h and sputtered with a gold layer, and viewed with Scanning Electron Microscope (Tescan VEGA3, China).

The bacteria pellets of strain YZUMF202001 were sent to BENAGEN Company (Wuhan, China) for whole genome sequencing. A genomic phylogram was constructed to accurately identify the strain using the reference genomes generated from GenBank and Ezbiocloud (https://www.ezbiocloud.net). The genome sequences were performed for gene prediction and single copy orthologue sequences. Then they were aligned and removed the non-informative columns of the resulting (concatenated) alignment. The phylogenetic tree was built by iqtree 2.1.4 (Nadal-Jimenez et al. 2022) and visualized by Figtree 1.4.4 (Rambaut 2012). Further, the identity was verified by calculating the average nucleotide identity (ANI) values using fastANI 1.3 (Jain et al. 2018), which is one of the most robust measurements of genomic relatedness between strains having great potential in the taxonomy of bacteria (Kim et al. 2014). The calculation results are visualized through the pheatmap software package in R (Kolde 2015).

### Nitrogen fixation ability of strain YZUMF202001

The strain YZUMF202001 and *Escherichia coli* DH5α were respectively cultured in LB broth at 28 ℃ for 12 h, these two strains were centrifuged and washed with sterile water for 3 times (8000 rpm, 10 min), adjusted to OD_600_ = 1, and inoculated 10 µL at each position on nitrogen-free culture medium for 5 days to test their NF ability (Liu et al. 2016). Each strain was transferred to Nfb medium (Dworkin et al. 2006) for acetylene reduction assay. The samples (0.4 mL Nfb liquid culture, OD_600_ = 1) inoculated in test tubes (100 mL) containing 40 mL Nfb liquid medium, and sealed with a silicone lid at 28 ℃ for 24 h. Controls were inoculated with *E. coli* DH5α using the same conditions. 10% (v/v) of the gas phase in test tubes was replaced with acetylene and kept for 24 h at 28 ℃. The gas was collected and sent to the public technology center of Institute of Soil Science, Chinese Academy of Sciences (Nanjing, China) for detection by gas chromatography-mass spectrometry system (MDGCMSMS 8050). Three replications were done for the test and the assay was repeated for two times. Data analysis was performed in Graphpad prism 8 (Zhou et al. 2022). The structural map of *nif* gene cluster for nitrogenase was drawn by SnapGene 6 0.2 (Altayb et al. 2022) and edited with Adobe Illustrator CS6.

### Restitution of symbiosis of GFP-labeled EHB

The plasmid pLac-EGFP-Chl-signal-Hyg purchased from Miaolingbio company (Figure S2) was transformed into *E. coli* S17-1 by heat shock method. Inoculate *E. coli* S17-1 containing GFP plasmid and *K. michiganensis* YZUMF202001 into fresh culture medium, in which the cells were grown at 37 ℃ to an OD_600_ of 0.6. Then, mix the strains S17-1 and YZUMF202001 in a 2:1 ratio (total volume 300 µL) centrifuge at 8000 rpm for 2 min and discard the supernatant. The bacteria were washed twice and resuspended in 30 µL of LB. The mixture were spread on LB plates and grown overnight at 30 ℃. The plates were washed with LB and 100 µl of the resulting suspension was plated on nitrogen free medium containing chloramphenicol (100 µg/ml). After growing in the dark at 28 ℃ for 2–3 days, detect the fluorescence of transformed YZUMF202001.

GFP labeled YZUMF202001 was inoculated in 50 mL LB containing chloramphenicol (100 µg/ml) and incubated at 28 ℃ for 12 h before observing using a fluorescence microscope. Host fungus YZZF202006 was inoculated in modified YEPSL medium (0.5% yeast extract, 1% peptone, 1% glucose, 0.1% MgSO_4_, 0.3% KH_2_PO_4_, 0.3 M CaCl_2_, 3000ppm polyoxin) and cultured at 28 ℃ for 3 days (We believe that improving the last two components of YEPSL to increase the permeability and abnormal deformation of the host cell wall would be more conducive to bacterial invasion of the host, and the concentration was determined after screening). Centrifuge 10 mL of YZZF202006 (8,000 rpm, 10 min), discard the supernatant, add 30 mL chitinase, glucanase, cellulase, and chitosan enzyme with a final concentration of 15 mg/ml (the enzymes were dissolved with sterilized PBS, and filtrated by 0.22 μm microporous filter, pH = 4–6) to resuspension the cell, and place it at 28 ℃ at 200 rpm for 4 h. The host fungi and GFP labeled bacteria were collected and washed twice with 0.8 M NaCl (8,000 rpm, 10 min). Both YZUMF202001 and host fungi YZZF202006 were resuspended with 0.8 M NaCl (OD600 = 1), then 100 µL of bacteria and 1 mL of the host fungus were inoculated to the modified minimal nutrient M9 medium (extra added 3000ppm polyoxin and 2% PTC solution) at a ratio of 1:10 and incubate them at 200 rpm at 37 ℃ for 3 days. Finally, spread 100 µL on the modified M9 plate containing hygromycin (100 µg/mL) and cephalosporin (50 µg/mL) for inhibiting the formation of bacterial colonies, and observe after the colony grows.

## Results

### Existence of EHB Klebsiella sp. in Ustilago maydis

**Fig. 1:**
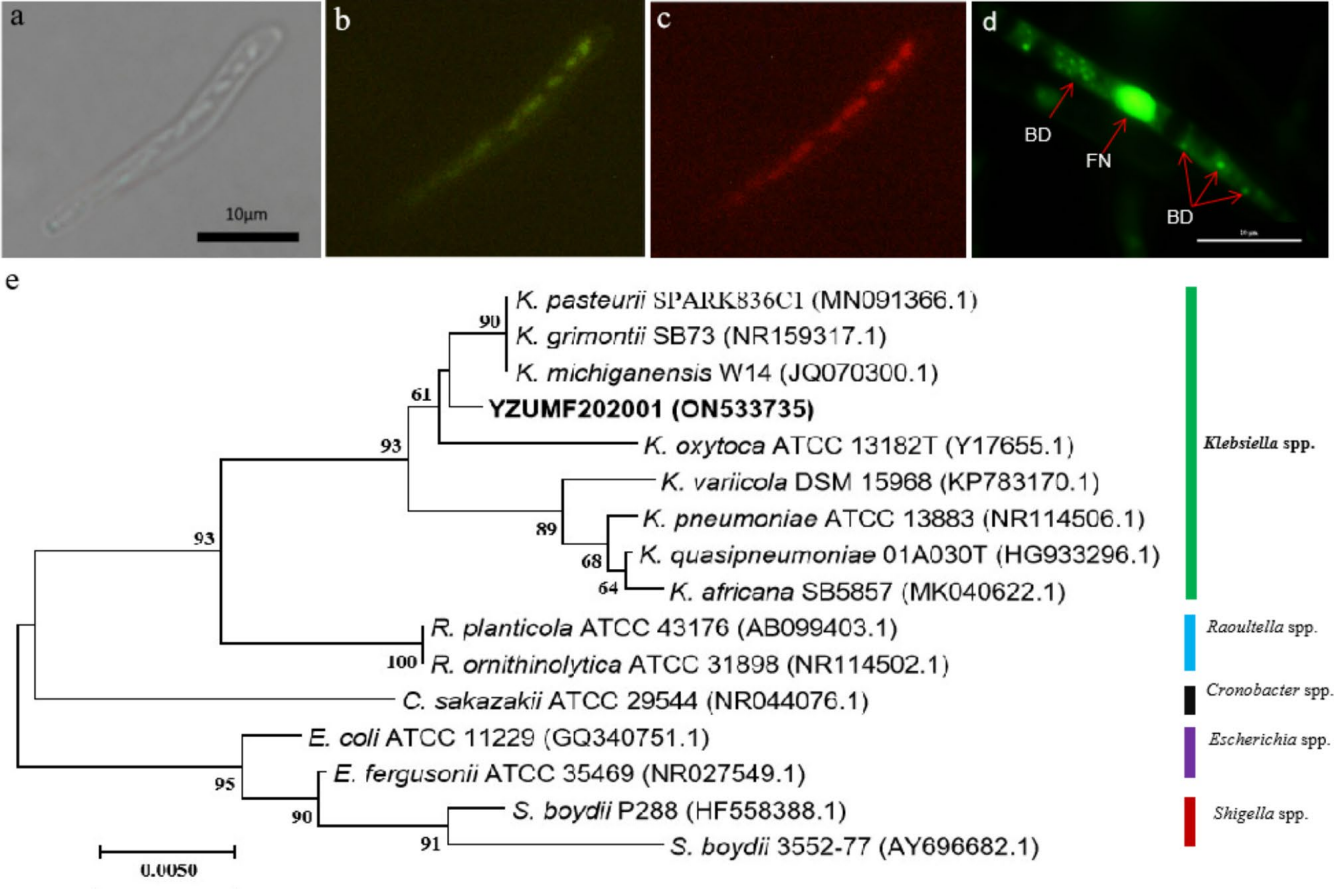
Fluorescence in situ hybridization (FISH) assay for *Ustilago maydis* YZZF202006 and phylogram of endohyphal bacterium YZUMF202001 (**a**) Cells observed in bright field; (**b**) Red fluorescent cells hybridized with Cy3; (**c**) Green fluorescent cells hybridized with FITC; (**d**) Stains of YZZF202006 with SYTO-9 green-fluorescent nucleic acid stains; FN: Fungal nucleus; BD: Bacterial DNA; (**e**): Maximum Likelihood tree for endohyphal bacterium *Klebsiella* sp. YZUMF202001 based on partial 16 S rRNA region

Microscopic observation showed that there were bacteria nuclei in hyphae of *U. maydis* YZZF202006 determined by fluorescence in situ hybridization (Fig. 1a-c). SYTO-9 is a fluorescent stain that can combine with nucleic acid in cells to produce strong fluorescence. It can be obviously observed that the nucleus of YZZF202006 was stained, at the same time, several bacterial like spots were observed in it, further proving that there are EHB in YZZF202006 (Fig. 1d). Phylogenetic analysis of partial 16s rDNA region resulted a 629 bp nucleotide sequence and deposited in GenBank with an accession number of ON533735. Several species of *Klebsiella* and other genera of Enterobacteriaceae (*Raoultella*, *Cronobacter*, *Escherichia* and *Shigella*) were selected to construct a phylogenetic tree. The ML tree revealed that strain YZUMF202001 fell into a clade of *Klebsiella* spp. with 93% bootstrap value support. The present strain was close to *K. pasteurii* and *K. grimontii* and *K. michiganensis*, but it could not be identified to species level (Fig. 1e). The results indicated that there was an EHB of *Klebsiella* sp. in *U. maydis* YZZF202006.

### Identification of EHB strain YZUMF202001

**Fig. 2:**
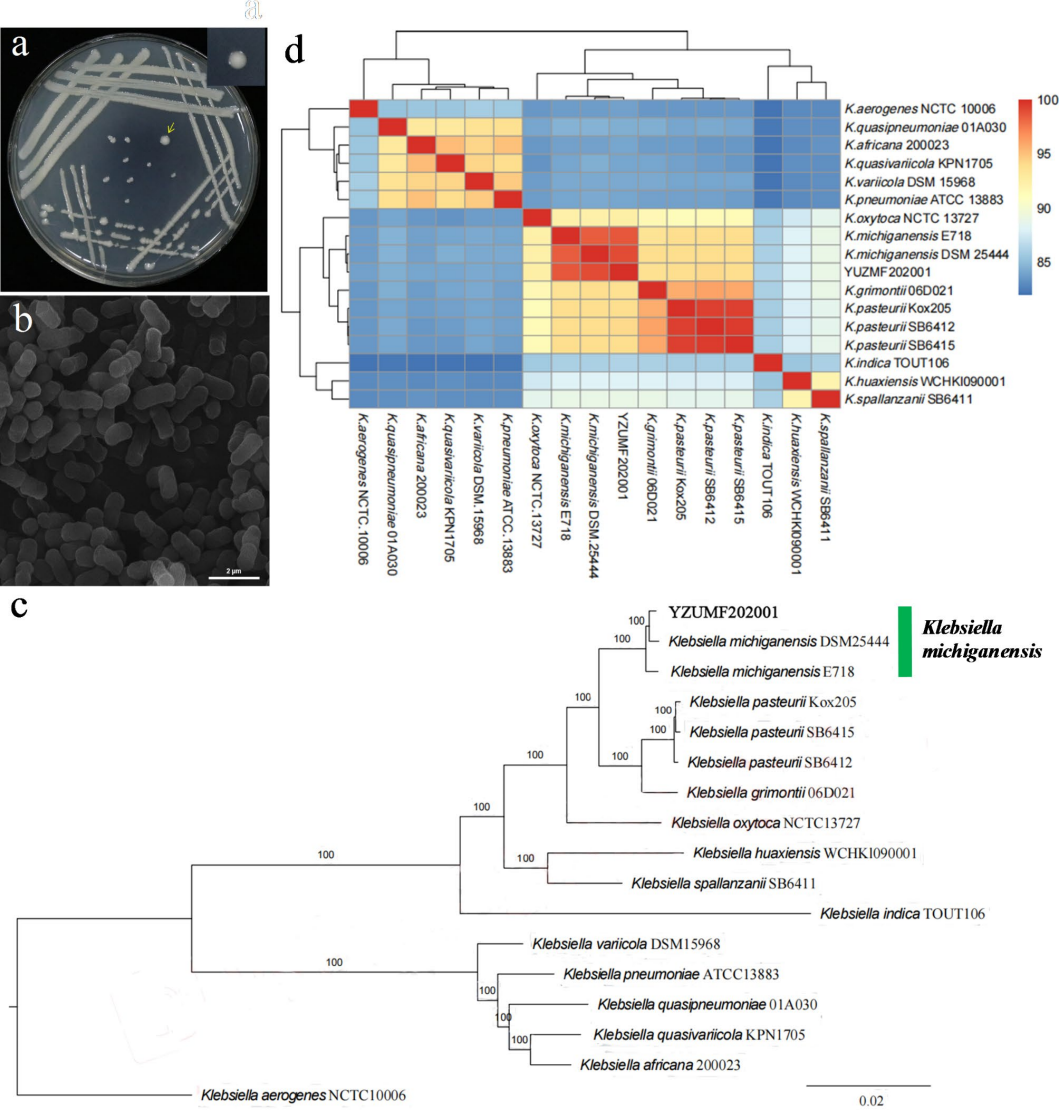
(**a**): Colony of endohyphal bacterium strain YZUMF202001 grown on LA meidium. (**b**): Bacterial cells of strain YZUMF202001 obtained by scanning electron microscope (SEM) observation. (**c**): Genomic phylogram of strain YZUMF202001 compared with relevant strains. (**d**): Heatmap of ANI analysis based on the genome sequences of 17 *Klebsiella* strains

The strain YZUF202001 formed circular, light yellow colony, smooth on the surface in LB plate (Fig. 2a), and determined as Gram-negative bacterium. The bacterial cells were short rods, with an average length of 1.5 μm and a width of around 0.5 μm. The exterior features were not smooth with slight wrinkles under scanning electron microscope (Fig. 2b). The general features of strain YZUMF202001 genome are shown in Table 1. The genome had uploaded to GenBank (Accession number CP097554). The genome circle map of YZUMF202001 was drawn (Figure S3). Based on the phylogram generated with single copy orthologue sequences, strain YZUMF202001 fell into *Klebsiella michiganensis* clade with 100% bootstrap values, but close to *K. michiganensis* DSM25444 by forming a subclade supported with 100% bootstrap values (Fig. 2c). The results showed that the present strain is *K. michiganensis*. Moreover, the ANI values (Table S1) were respectively 99.04% (*K. michiganensis* DSM25444) and 98.68% (*K. michiganensis* E718) when compared between strain YZUMF202001 and the two strains of *K. michiganensis*. It was less than 95% when compared with other *Klebsiella* species (Fig. 2d). The ANI values of 95–96% can be used as a boundary for species delineation (Goris et al. 2007; Amann 2001). The ANI analysis also verified that strain YZUMF202001 was *K. michiganensis*.

**Table 1:** Genomic information statistics of *K. michiganensis* YZUMF202001

Features	YZUMF202001
Contig number	1
Contig Length (bp)	6,048,476
Contig N50 (bp)	6,048,476
GC content(%)	56.01
Gaps Number	0
Gene	5753
CDS	5506
tRNA	85
rRNA	25
tmRNA	1

### Nitrogen fixation ability of YZUMF202001

**Fig. 3:**
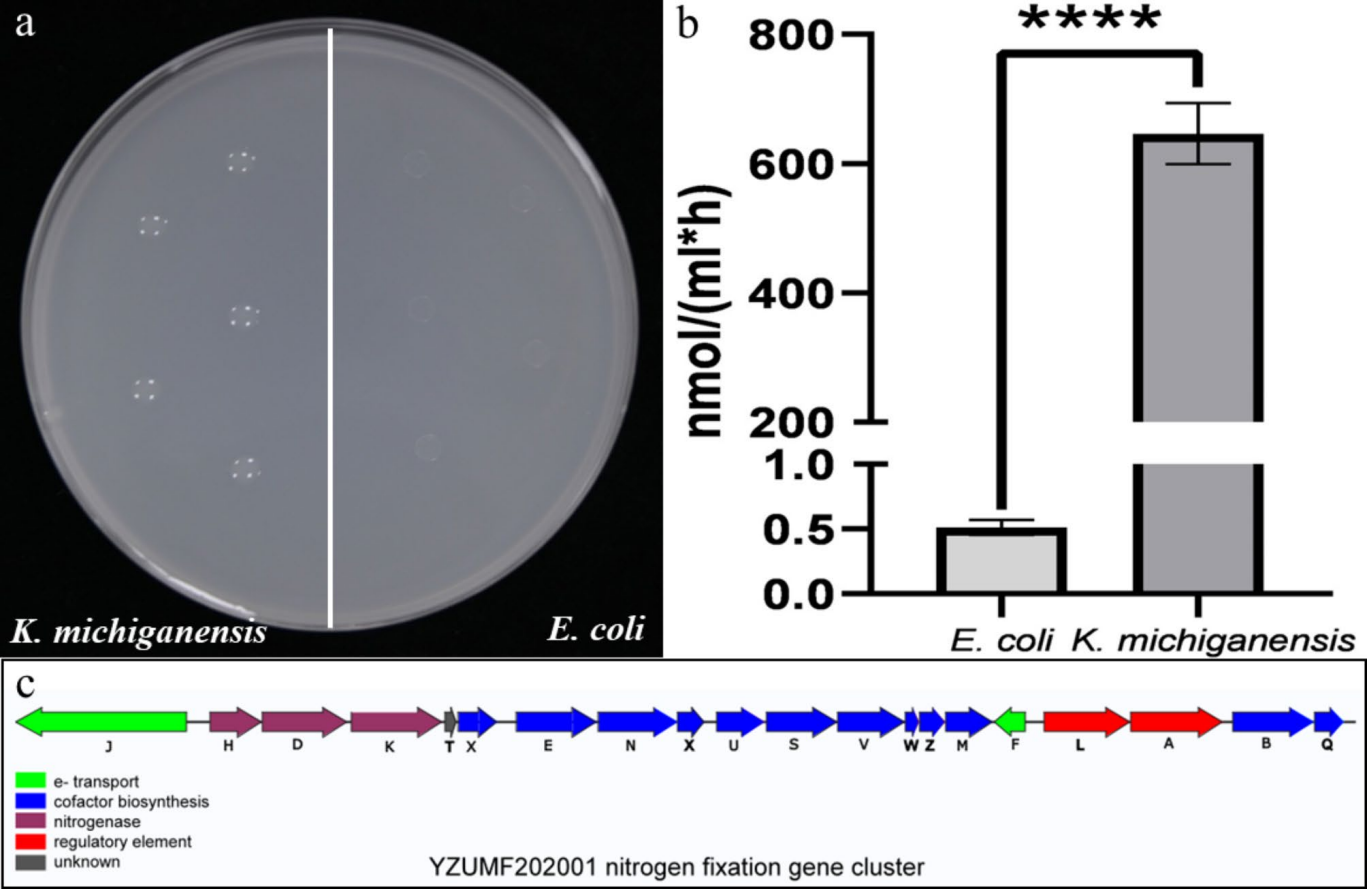
(**a**) Preliminary nitrogen fixation capacity test with nitrogen-free culture medium (left: strain YZUMF202001; right: strain DH5α), (**b**) The value of nitrogenase activity determined by acetylene reduction assay (left: strain DH5α; right: strain YZUMF202001), (**c**) nitrogen fixation gene cluster of strain YZUMF202001

Both strain YZUMF202001 and *Escherichia coli* DH5α were pointed cultured on nitrogen-free plates. After 5 days, the colonies of YZUMF202001 occurred and grown well with smooth and glossy surface, while *E. coli* DH5α could not grow normally (Fig. 3a). It determined that the EHB strain YZUMF202001 had the ability of NF. Its value of nitrogenase activity was av. 646.25 ± 38.61 nmol·mL^− 1^·h^− 1^ C_2_H_4_, which significantly different from *E. coli* DH5α (****: *P* < 0.0001) (Fig. 3b). The structure map of nitrogen fixation gene cluster was drawn according to the gene annotation results of strain YZUMF202001 (Fig. 3c), which consisted of 20 genes (*nifJHDKTXENXUSVWZMFLABQ*) encoded in 23.5 kb of DNA. It is marked with different colors depending on their different functions, which is similar to those of *Klebsiella oxytoca* and *K. peneumoniae* (Temme et al. 2012; Arnold et al. 1988).

### Restitution of symbiosis of GFP-labeled EHB

**Fig. 4:**
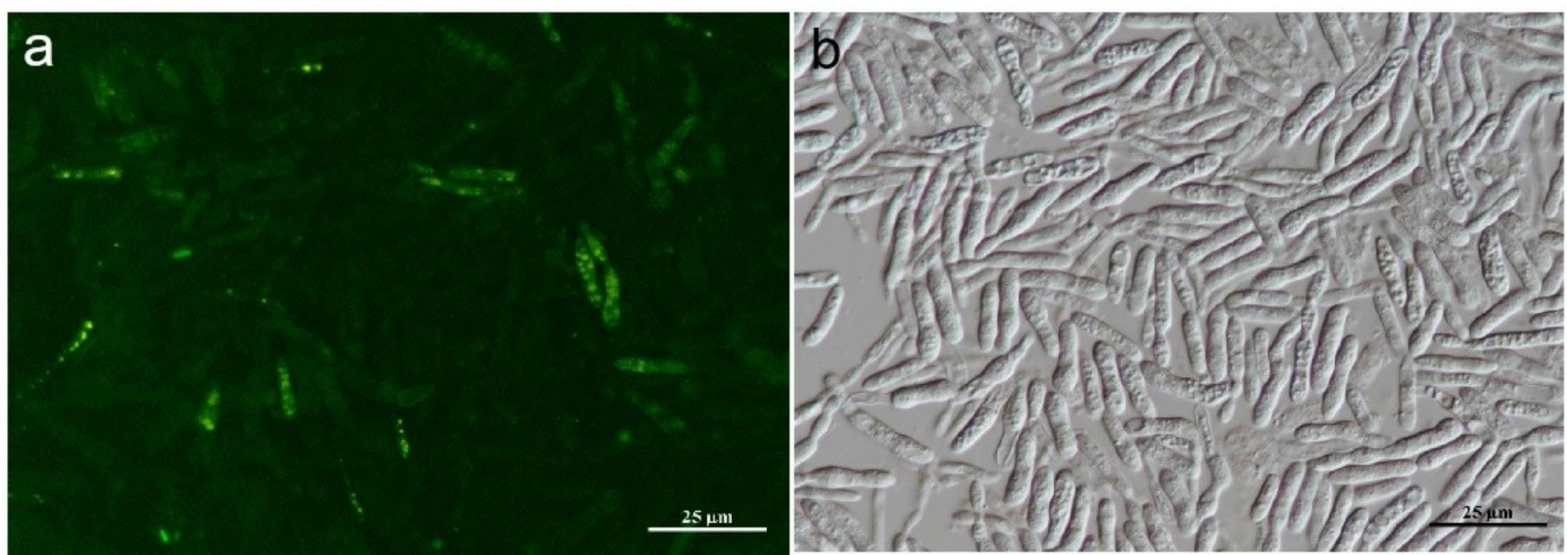
GFP-labeled YZUMF202001 colonizing YZZF202006 was observed in fluorescence (**a**) and white light (**b**) mode, scale bar represents 25 μm

In order to test the hypothesis that *K. michiganensis* was capable of reintroducing *U. maydis*, several methods have been tried. We cocultured a GFP-labeled *K. michiganensis* and *U. maydis* for 72 h, and also cocultured MgCl_2_ treated fungi and EHB on plates, but the results were not satisfactory (Spraker et al. 2016; Arendt et al. 2016). EHB was successfully reintroduced into the original host fungus after treatment as described in the method (Fig. 4). Fluorescence microscope confirmed that a substantial number of bacteria were localized to the intracellular space of some *U. maydis*. The GFP-labeled EHB in the *U. maydis* is spherical, which is different from that in the free-living (short rod). This research result is consistent with the difference in the shape of *Enterobacter* sp. in and out of the hyphae (Obasa et al. 2017). Unfortunately, only about one tenth of *U. maydis* were reintroduced by the GFP-labeled EHB, which was not very effective.

## Discussion

Previous studies have shown that there are different endohyphal bacteria living in the hyphae of various fungi. Liu et al. (2019) summarized the fungal hosts associated with endohyphal bacteria (EHB), which included 4 genera in *Mucoromycota*, 7 in *Glomeromycota*, 13 in *Asomycota* and 13 in *Basidiomycota* (Liu et al. 2019). The results indicate that EHBs are commonly found in the cells of *Ascomycota* and *Basidiomycota*. As a *Basidiomycota*, *Ustilago maydis* has been determined containing EHB *Bacillus* spp. without obtain the bacterial strains (Ruiz-Herrera et al. 2015). Similarly, there were many bacteria observed in the hyphae of *U. maydis* in the present study. A bacterial strain YZUMF202001 of *Klebsiella michiganensis* was successfully isolated from protoplasts of *U. maydis* hyphae, which is first report of a cultural EHB from *U. maydis* worldwide.

Around 60 bacterial genera as EHB have been found associated with fungi (Liu et al. 2019). Among them, *Alphaproteobacteria* and *Betaproteobacteria* are predominant endohyphal species. However, a few species had been reported from *Gammaproteobacteria*, such as *Pseudomonas* spp., *Serratia* spp. and *Klebsiella* spp. For the genus *Klebsiella*, *K. pneumoniae* is found as endohyphal bacterium collected from *Rhizopus oryzae* (generated from wheat starch and bread), which is an opportunistic pathogen dangerous for human (Birol and Gunyar 2021). In the other hand, an EHB *K. aerogenes* is isolated from an endophytic *Fusarium oxysporum* of *Bletilla striata*, which EHB produces IAA enhancing the growth promoting ability of the host fungi (Cheng et al. 2022). In the study, *K. michiganensis* is a new EHB *Klebsiella* species, enriching the diversity of EHB.

*Klebsiella* spp. are correlated with corn or its rhizosphere, which has been found from corn roots and the rhizosphere soil collected from five areas in South Brazil (Arruda et al. 2013). *K. oxytoca* is isolated from the rhizosphere of inbred corn plant var. Bisma in Indonesia (Setyowatia et al., 2017). A high level of *Klebsiella* colonization was observed in the rhizosphere soil of corn seedlings (Yang and Yang 2020). In Guangzhou of China, *K. michiganensis* is reported from corn rhizosphere soil (Long et al. 2017).*U. maydis* can overwinter on the diseased plant residues existed in field or soil, and can infect all green parts of plant, including stems, leaves, tassels and ears. It provides the great opportunity for the correlation between *Klebsiella* spp. and *U. maydis*, which probable occurs a bacterial invasion event (Bolker 2001; Bonfante and Desiro 2017) resulting in the presence of EHB *Klebsiella* spp. in *U. maydis*. In addition, chitin is the important cell wall component for fungi, and the type II secretion system (T2SS) of bacteria releases chitinase that is necessary for bacteria to enter into hyphae (Moebius et al. 2014). For the *K. michiganensis* strain YZUF202001 does not contain T2SS, but it contains chitinase related genes to help enter into hyphae of *U. maydis* (Table S2). The importance of chitinase is also shown in the restitution of EHB symbiosis. We also added several enzymes other than chitinase to help EHB dissolve the cell wall of the fungal host and enter its body (Gazzanelli et al. 1999; Filippi et al. 1998). In addition, the restitution of EHB symbiosis is also inseparable from low nutrition strategies, such as 1/4 PDB and M9 (Obasa et al. 2017, 2019; Baltrus et al. 2018; Hazarika et al. 2020; Cheng et al. 2022). The above two strategies match the methods used in this experiment.

*Ustilago maydis* can be able to grow normally in nitrogen-free medium and evidenced containing endohyphal bacteria with NF (Ruiz-Herrera et al. 2015). It is re-verified in the present study. Under the control conditions, the amount of ethylene from acetylene reduction is directly correlated to the nitrogenase activity (Palus et al. 1996). The nitrogenase activity is examined by acetylene reduction assay using EHB comprising fungi, such as *Tuber magnatum* (0.5–7.5 µmol C_2_H_4_ h^− 1^ g^− 1^) (Barbieri et al. 2010), R. *mucilaginosa* JGTA-S1 (0.2945 ± 0.0363 nmol of ethylene / h) (Paul et al. 2020)d *maydis* (57.84 ± 0.16 nmoles mg^− 1^ protein) (Ruiz-Herrera et al. 2015). Since the amount and taxonomy of EHBs in the host is uncertain and different, so the nitrogenase activity cannot be used for comparison. Fortunately, the EHB of *Klebsiella michiganensis* was isolated from *U. maydis* and its nitrogenase activity was determine accurately (646.25 ± 38.61 nmol·mL^− 1^·h^− 1^ C_2_H_4_). It sets a example for examining the NF ability of EHB.

Nitrogenase is essential for the biological nitrogen fixation, composing two proteins, dinitrogenase (*nifD* and *nifK* genes products) and dinitrogenase reductase (*nifH* gene product) (Rubio and Ludden 2005). It also includes cofactor biosynthesis and operons encoded by other *nif* related genes (Temme et al. 2012). The reported nitrogenases of *Klebsiell*a spp. are generally Mo-nitrogenase, which is the most well-studied with the highest NF ability compared with other nitrogenase (Sickerman et al. 2019). In this study, the *nif* gene structure of *K. michiganensis* YZUMF202001 is consistent to those reports of *K. oxytoca* and *K. peneumoniae* (Temme et al. 2012; Arnold et al. 1988). *Klebsiella michiganensis* stimulates the growth of maize grains considered to be the best maize growth promoting bacteria (Elsayed et al. 2019), which can produce indole-3-acetic acid (IAA) and siderophore (Long et al. 2017). *Klebsiella variicola* has a variety of biological functions including nitrogen fixation, and the species can colonize in corn rhizosphere soil and promote the growth of the seedlings (Yang and Yang 2020). The IAA and siderophore production and the plant promoting ability will be further conducted for the present strain YZUMF202001.

### Electronic supplementary material

Below is the link to the electronic supplementary material.

Supplementary Material 1

## Data Availability

All data generated during this study are included in this published article (and its supplementary material file). *Klebsiella michiganensis* YZUMF202001 strain have stored in China Typical Culture Collection Center (WDCM number 611) (preservation number: CCTCC M 2,022,708).
