# Supplementary material for: Klebsiella michiganensis: a nitrogen-fixing endohyphal bacterium from Ustilago maydis
Source: AMB Express. 2023 Dec 19;13:146. doi: 10.1186/s13568-023-01618-8 (PMC10730499; doi:10.1186/s13568-023-01618-8)
Supplement: Supplementary file 1 — Supplementary Material 1 [file 13568_2023_1618_MOESM1_ESM.docx]

AMB Express

***Klebsiella michiganensis*: A nitrogen-fixing endohyphal bacterium from *Ustilago maydis***

Pengyu Liang^1^, Jianwei Jiang^1^, Zhengxiang Sun^1^, Yanyan Li^2^, Chunlei Yang^2*^, Yi Zhou^1*^

^1^Department of Plant Protection, College of Agriculture, Yangtze University, Jingzhou 434025, China

^2^Tobacco Research Institute of Hubei Province, Wuhan 430000, China

*Corresponding author. Tel: +86-13617263127; Fax: +86-13617263127

E-mail address: [zhouyi@yangtzeu.edu.cn](mailto:zhouyi@yangtzeu.edu.cn) (Y. Zhou); ycl193737@163.com(C. Yang)

Figure S1. (a): Colony of strain *Ustilago* *maydis* YZZF202006 grown on YEPSL medium. (b): Haploid primary hypha. (c): Phylogenetic tree (Maximu likelihood) of strain *Ustilago* *maydis* YZZF202006 based on the ITS region. Bootstrap values (≥60%) of a bootstrap test of 1,000 replicates are shown on branching points. The GenBank accession number are noted after the strains names.


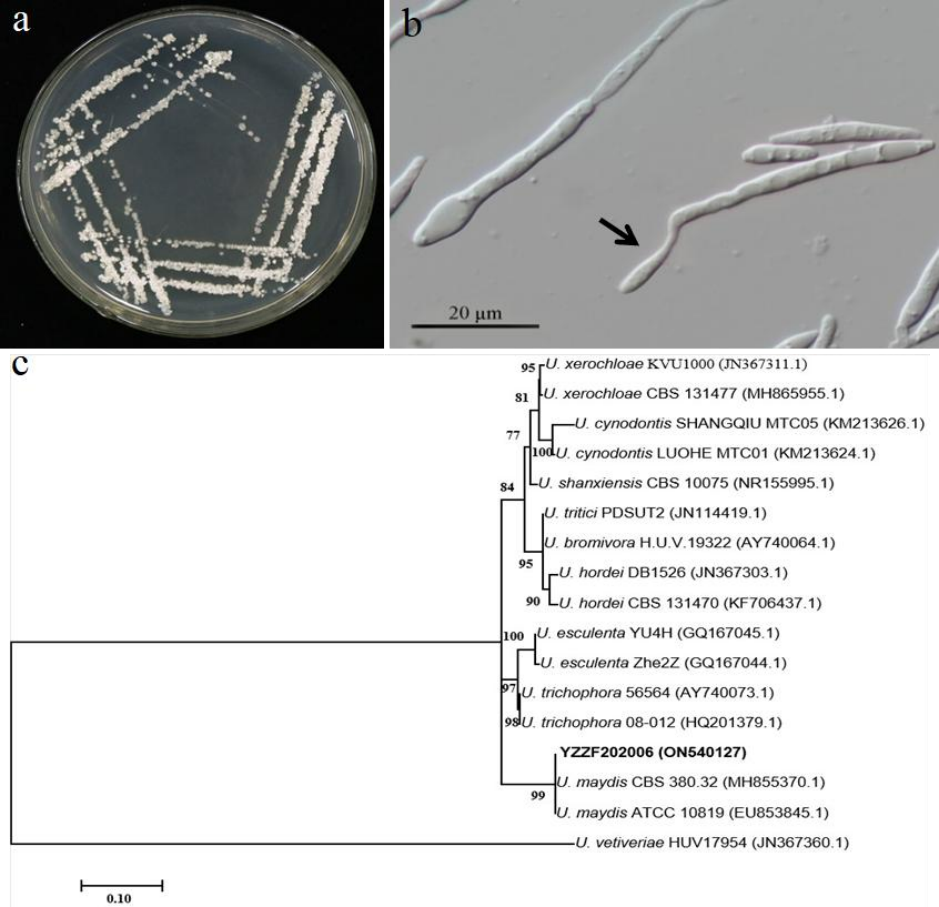


Figure S2. Map of plasmid pLac-EGFP-Chl-signal-Hyg (Miaolingbio, China).


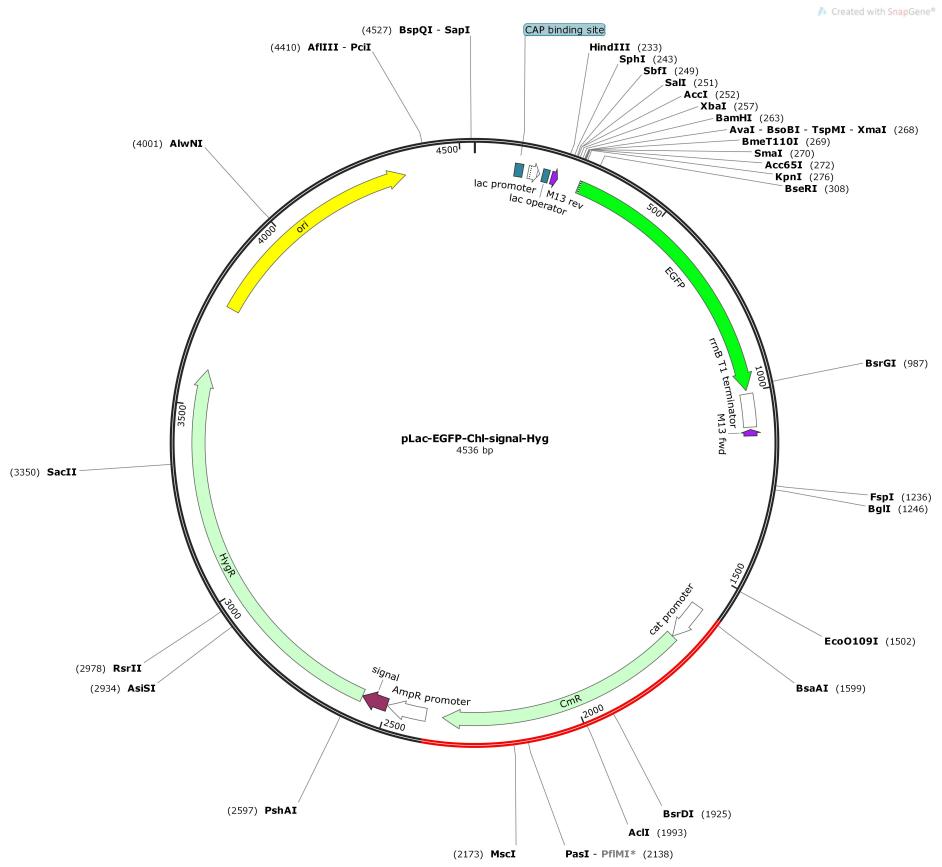


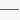

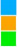
Figure S3. The circular genome map of *Klebsiella* *michiganensis* YZUMF202001.

chr1

−0. 1

0kb

0

100

50

150

200

100

0

200

300

400

500

0

0. 1

0.2

65

60

55

50

45

40

35


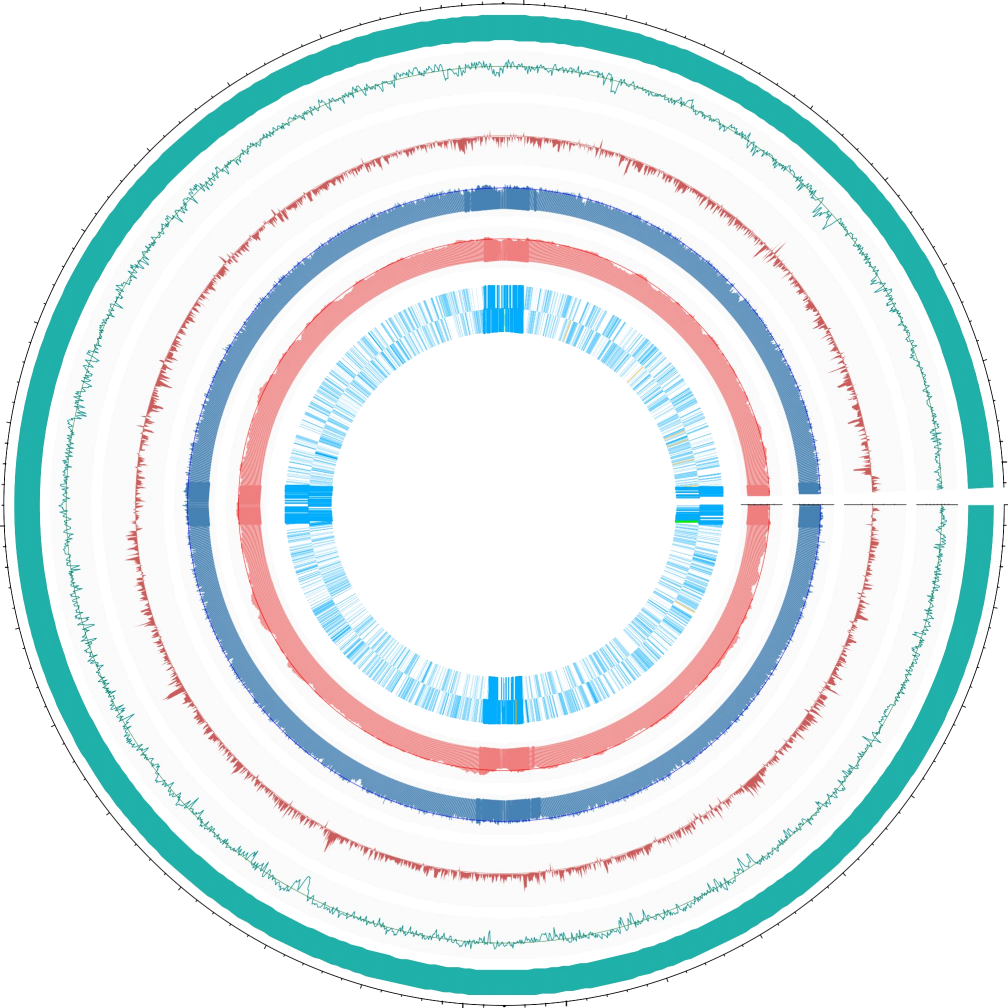
Refer size: 6048476 bp

Refer GC: 56.01

GC % ( Average: 56.01 % )


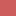
 GC shew

Coverage of illumina: 99.9999669338194 %


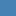
 Depth of illumina ( average: 453. 15 X )

Coverage of nanopore: 99.9996693381936 %


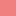
 Depth of nanopore ( average: 165.89 X )

CDS | rRNA | tRNA

CDS

rRNA

tRNA

**Table S1: Ani values of 17 *Klebsiella* strains including YZUMF202001 based on genome information.**

|  | K.aerogenes NCTC 10006 | K.africana 200023 | K.huaxiensis WCHKl090001 | K.indica TOUT106 | K.pasteurii SB6412 | K.spallanzanii SB6411 | K.grimontii 06D021 | K.michiganensis DSM 25444 | K.oxytoca NCTC 13727 | K.variicola DSM 15968 | K.pneumoniae ATCC 13883 | K.quasipneumoniae 01A030 | K.quasivariicola KPN1705 | **YZUMF202001** | K.pasteurii Kox205 | K.pasteurii SB6415 | K.michiganensis E718 |
| --- | --- | --- | --- | --- | --- | --- | --- | --- | --- | --- | --- | --- | --- | --- | --- | --- | --- |
| K.aerogenes NCTC 10006 | 100 | 85.49 | 82.91 | 82.1 | 83.44 | 83 | 83.54 | 83.71 | 83.51 | 85.83 | 85.59 | 85.22 | 85.43 | **83.82** | 83.42 | 83.48 | 83.67 |
| K.africana 200023 | 85.49 | 100 | 82.89 | 81.92 | 83.46 | 83.21 | 83.66 | 83.87 | 83.53 | 94.29 | 95.1 | 93.06 | 95.15 | **83.95** | 83.65 | 83.45 | 83.88 |
| K.huaxiensis WCHKl090001 | 82.91 | 82.89 | 100 | 85.43 | 87.71 | 91.93 | 87.94 | 87.97 | 87.46 | 82.97 | 82.84 | 83.1 | 83.09 | **87.95** | 87.74 | 87.74 | 87.91 |
| K.indica TOUT106 | 82.1 | 81.92 | 85.43 | 100 | 85.85 | 85.99 | 85.94 | 86.07 | 85.57 | 82.08 | 82.14 | 82.24 | 82.06 | **86.09** | 85.81 | 85.87 | 85.97 |
| K.pasteurii SB6412 | 83.44 | 83.46 | 87.71 | 85.85 | 100 | 88.59 | 96.03 | 94 | 91.33 | 83.94 | 83.78 | 84.09 | 83.56 | **94.04** | 99.43 | 99.48 | 93.88 |
| K.spallanzanii SB6411 | 83 | 83.21 | 91.93 | 85.99 | 88.59 | 100 | 88.86 | 88.93 | 88.52 | 83.12 | 83.03 | 83.18 | 83.16 | **88.93** | 88.5 | 88.69 | 88.83 |
| K.grimontii 06D021 | 83.54 | 83.66 | 87.94 | 85.94 | 96.03 | 88.86 | 100 | 93.86 | 91.51 | 84.13 | 83.87 | 84.42 | 83.98 | **93.85** | 95.82 | 95.94 | 93.6 |
| K.michiganensis DSM 25444 | 83.71 | 83.87 | 87.97 | 86.07 | 94 | 88.93 | 93.86 | 100 | 92.38 | 84.25 | 84.1 | 84.47 | 84.33 | **99.04** | 93.91 | 93.97 | 98.44 |
| K.oxytoca NCTC 13727 | 83.51 | 83.53 | 87.46 | 85.57 | 91.33 | 88.52 | 91.51 | 92.38 | 100 | 83.67 | 83.59 | 83.91 | 83.32 | **92.44** | 91.26 | 91.38 | 92.35 |
| K.variicola DSM 15968 | 85.83 | 94.29 | 82.97 | 82.08 | 83.94 | 83.12 | 84.13 | 84.25 | 83.67 | 100 | 94.66 | 93.64 | 94.22 | **84.24** | 83.97 | 83.97 | 84.22 |
| K.pneumoniae ATCC 13883 | 85.59 | 95.1 | 82.84 | 82.14 | 83.78 | 83.03 | 83.87 | 84.1 | 83.59 | 94.66 | 100 | 93.71 | 93.97 | **84.01** | 83.82 | 83.7 | 83.95 |
| K.quasipneumoniae 01A030 | 85.22 | 93.06 | 83.1 | 82.24 | 84.09 | 83.18 | 84.42 | 84.47 | 83.91 | 93.64 | 93.71 | 100 | 93.03 | **84.34** | 83.97 | 83.99 | 84.56 |
| K.quasivariicola KPN1705 | 85.43 | 95.15 | 83.09 | 82.06 | 83.56 | 83.16 | 83.98 | 84.33 | 83.32 | 94.22 | 93.97 | 93.03 | 100 | **83.94** | 83.75 | 83.41 | 83.99 |
| **YUZMF202001** | **83.82** | **83.95** | **87.95** | **86.09** | **94.04** | **88.93** | **93.85** | **99.04** | **92.44** | **84.24** | **84.01** | **84.34** | **83.94** | **100** | **93.98** | **94.03** | **98.68** |
| K.pasteurii Kox205 | 83.42 | 83.65 | 87.74 | 85.81 | 99.43 | 88.5 | 95.82 | 93.91 | 91.26 | 83.97 | 83.82 | 83.97 | 83.75 | **93.98** | 100 | 99.43 | 93.86 |
| K.pasteurii SB6415 | 83.48 | 83.45 | 87.74 | 85.87 | 99.48 | 88.69 | 95.94 | 93.97 | 91.38 | 83.97 | 83.7 | 83.99 | 83.41 | **94.03** | 99.43 | 100 | 93.94 |
| K.michiganensis E718 | 83.67 | 83.88 | 87.91 | 85.97 | 93.88 | 88.83 | 93.6 | 98.44 | 92.35 | 84.22 | 83.95 | 84.56 | 83.99 | **98.68** | 93.86 | 93.94 | 100 |

**Table S2: Chitinase related genes of *Klebsiella michiganensis* YZUMF202001.**

| Gene | Description | E.C. number | Chromosome location | Gene_id |
| --- | --- | --- | --- | --- |
| chiA1 | Chitinase A1 | 3.2.1.14 | 3563132-3564385 | assembly_03407 |
| chiA_1 | Chitinase A | 3.2.1.14 | 4358697-4360814 | assembly_04169 |
| chiA_2 | Chitinase A | 3.2.1.14 | 4917333-4919123 | assembly_04693 |
| chiA_3 | Chitinase A | 3.2.1.14 | 5157108-5159207 | assembly_04898 |
